# Supplementary material for: Integrated healthy lifestyle even in late-life mitigates cognitive decline risk across varied genetic susceptibility
Source: Nat Commun. 2025 Jan 9;16:539. doi: 10.1038/s41467-024-55763-0 (PMC11718162; doi:10.1038/s41467-024-55763-0)
Supplement: Supplementary file 1 — Supplementary Information [file 41467_2024_55763_MOESM1_ESM.pdf]

Supplementary information

Integrated healthy lifestyle even in late-life mitigates cognitive decline risk across varied genetic susceptibility

Jun Wang, PhD [1,2], Chen Chen, PhD [1,2], Jinhui Zhou, PhD [1,3], Zinan Xu, MBBS [1,4], Lanjing Xu, MMed [1,5], Xinwei Li, PhD [1,6], Zhuchun Zhong, MBBS [1,7], Yuebin Lv, PhD [1,2*], and Xiaoming Shi, PhD [1,2*]

Author affiliations

[1]China CDC Key Laboratory of Environment and Population Health, National Institute of Environmental Health, Chinese Center for Disease Control and Prevention, Beijing, China

[2]National Key Laboratory of Intelligent Tracking and Forecasting for Infectious Diseases, National Institute of Environmental Health, Chinese Center for Disease Control and Prevention, Beijing, China

[3]National Cancer Center/National Clinical Research Center for Cancer/Cancer Hospital, Chinese Academy of Medical Sciences and Peking Union Medical College, Beijing, China

[4]Department of Epidemiology, School of Public Health, Southern Medical University, Guangzhou, Guangdong, China

[5]Department of Big Data in Health Science, School of Public Health, Zhejiang University, Hangzhou, Zhejiang, China

[6]Department of Epidemiology and Biostatistics, School of Public Health, Jilin University, Changchun, Jilin, China

[7]Institute of Environmental Medicine, Zhejiang University School of Medicine, Hangzhou, Zhejiang, China

[*]These authors jointly supervised this work: Yuebin Lv, and Xiaoming Shi

Supplementary Methods

Assessment of healthy lifestyle factors

Smoking status

Smoking status was assessed at baseline through the following questions "do you smoke at present", "did you smoke in the past". We classified smoking status as never smoking, former smoking and current smoking. Current non-smoking (never smoking, or former smoking) was deemed a healthy lifestyle factor and allocated a score of 1[1, 2].

Alcohol drinking status

Alcohol drinking status was evaluated using the following four questions: "do you drink alcohol at the present", "did you drink alcohol in the past". We classified alcohol drinking status as never drinking, former drinking and current drinking. Never alcohol drinking was deemed a healthy lifestyle factor and allocated a score of 1[1, 2].

Physical activity

Physical activity was assessed by frequency of 9 different activities: regular exercises, housework tasks, personal outdoor activities, gardening, rearing domestic animals/pets, reading, playing cards/mahjong, watching TV/listening to the radio, and attending social activities, with five response options. Personal outdoor activities referred to individuals leaving their familiar house to participate in positive activities, such as Tai Chi, square dancing, and other outdoor activities[3]. Social activities were defined as activities that involved communication with others and included, such as, participating in volunteer work or organized group discussions[4, 5]. For each activity was coded as follows: "almost every day/not every day, but once for a week" (coded: 2), "not every week, but at least once for a month" (coded: 1), and "not every month, but sometimes/never" (coded: 0) according to previous studies[6, 7, 8]. For each participant, these values (0, 1, or 2) were summed up across the 9 activities to a total physical activity score (range 0–18). Ideal physical activity was defined as the top 40% of the cohort distribution in line with previous study[9].

Diet intake

A validated simplified food frequency questionnaire (FFQ) was used for assessing dietary information[10, 11, 12]. Participants were asked to report their current food frequencies for intake of 9 major foods (fresh vegetables, fresh fruit, legumes, meat, egg, fish and seafood, salty vegetables, tea, and garlic). Salty vegetables, as a Chinese traditional food, are often preserved in soy sauce or salt for a long time[13]. For fresh vegetables and fresh fruit consumption, the answer of "everyday or almost everyday/quite often", "occasionally", "rarely or never" received 2, 1, or 0, respectively. For salty vegetables, the answer of "almost every day/not every day but at least once per week", "not every week but at least once per month", "not every month but occasionally/ rarely or never" received 0, 1, or 2, respectively. For the other 6 foods, the same three responses received scores of 2, 1, or 0, respectively. Scores for the 9 foods were then summed to obtain a scale ranging from 0 to 18[11]. Ideal diet intake was defined as the top 40% of the cohort distribution in line with previous studies[2, 14].

The four lifestyle factors scores were then summed to yield a final healthy lifestyle

score ranging from 0 to 4, with higher values representing a healthier lifestyle. Table S1 presents the categories and scores for the individual lifestyle components.

Assessment of covariates

Age was calculated by using the birth date and the date of baseline assessment. Sex data were self-reported using structured questions, with the options “female” or “male” provided. The educational level was based on self-reported years of schooling and further categorized as “no formal education (educational attainment <1 year)”, “elementary school level (1–6 years of schooling)” or “high school level or above (more than 6 years of schooling)”. Participants’ area of residence was categorized as residing in rural or urban areas. Marital status was categorized as “not in marriage” if a participant had never married or was widowed or divorced and “in marriage” if a participant was currently married. Occupation was divided into “agriculture/forestry/ husbandry/fishery”, “commercial, service, industrial worker/self-employed”, “professional/governmental/managerial personnel”, or “houseworker/never worked/other[15]. Source of income was classified as “independent” or “dependent” based on response to the question “do you have your own income?”. Health status was based on self-reported health status, and divided into two groups “poor” or “good”. Optimism status was measured based on the following 7 items on 5-point response scale (“all of the time”, “frequently”, “sometimes”, “rarely”, and “never”): (1) looking on the bright side of things, (2) keeping your belongings neat and clean, (3) making your own decisions concerning your personal affairs, (4) feeling as happy as you are when you are young, (5) feeling nervous and scared, (6) feeling lonely and isolated, (7) thinking that the older you are, the less useful you are, and the hard work you do. Participants were assigned one point for each item (1= “always/often/sometimes”, 0= “seldom/never”). Some questions were asked about the opposite status, and we reverse-assigned the interview’s answer. Therefore, all items were summed to create a composite score that ranged from 0–7, with higher scores indicating higher levels of optimism[16]. A history of major chronic diseases including cardiovascular disease, diabetes mellitus, hypertension, respiratory disease, digestive system disease, or cancer was collected.

Supplementary Table 1. Rate of change in cognitive score according to genetic risk

Genetic risk	Standard deviation units			Difference, %
	Estimate (95% CI)	Difference (95% CI)	*P* value	
Cognitive score				
High genetic risk	−0.184 (−0.199, −0.169)	Reference		Reference
Low genetic risk	−0.161 (−0.176, −0.146)	0.023 (0.003, 0.044)	0.025	12.5
Orientation score				
High genetic risk	−0.177 (−0.194, −0.160)	Reference		Reference
Low genetic risk	−0.152 (−0.169, −0.135)	0.025 (0.002, 0.049)	0.036	14.12
Attention and calculation score				
High genetic risk	−0.074 (−0.082, −0.067)	Reference		Reference
Low genetic risk	−0.066 (−0.073, −0.058)	0.009 (−0.002, 0.019)	0.102	12.16
Visual construction score				
High genetic risk	−0.003 (−0.008, 0.003)	Reference		NA
Low genetic risk	0.000 (−0.005, 0.006)	0.003 (−0.004, 0.011)	0.406	
Language score				
High genetic risk	−0.137 (−0.150, −0.123)	Reference		Reference
Low genetic risk	−0.113 (−0.127, −0.100)	0.023 (0.005, 0.042)	0.014	16.79
Naming score				
High genetic risk	−0.059 (−0.067, −0.050)	Reference		Reference
Low genetic risk	−0.048 (−0.056, −0.039)	0.011 (−0.001, 0.023)	0.063	18.64
Recall score				
High genetic risk	−0.078 (−0.087, −0.069)	Reference		Reference
Low genetic risk	−0.066 (−0.075, −0.058)	0.012 (−0.000, 0.023)	0.055	15.38

CI confidence interval.

Linear mixed-effects models were used with adjustment for age, sex, entry time, educational attainment, area of residence, current marital status, occupation, source of income, and baseline cognitive score. For the analysis of six cognitive dimensions, models were additionally adjusted for the baseline dimensions of cognitive score as appropriate instead of baseline cognitive score. Two-sided $P < 0.05$ was considered statistically significant, except separate analysis for individual domains of cognition in which the Bonferroni correction was applied to account for multiple testing ($P < 0.008$ considered significant [= 0.05/6]).

Supplementary Table 2. Association between lifestyle categories and genetic risk and risk of cognitive impairment

Characteristics	Total No. of participants	No. of cases of cognitive impairment/person-years	Cox proportional hazard models*			Competing risk method based on Cox model#		
			Hazard ratio (95% CI)	*P* value	*P* trend	Hazard ratio (95% CI)	*P* value	*P* trend
Overall participants								
Lifestyle category					<0.001			<0.001
Unfavorable lifestyle	10515	3649/63265	Reference			Reference		
Intermediate lifestyle	5885	1714/40110	0.85 (0.80–0.90)	<0.001		0.90 (0.85–0.96)	<0.001	
Favorable lifestyle	2411	470/18271	0.69 (0.62–0.76)	<0.001		0.75 (0.68–0.83)	<0.001	
Participants with genetic information								
Lifestyle category					<0.001			0.013
Unfavorable lifestyle	3111	910/24075	Reference			Reference		
Intermediate lifestyle	2146	563/17678	0.90 (0.81–1.01)	0.069		0.94 (0.84–1.05)	0.271	
Favorable lifestyle	1044	183/8956	0.76 (0.64–0.89)	0.001		0.80 (0.68–0.95)	0.01	
Genetic risk category								
Low genetic risk	3150	806/25431	Reference		NA	Reference		NA
High genetic risk	3151	850/25278	1.10 (1.00–1.21)	0.043		1.08 (0.98–1.20)	0.129	

CI confidence interval.

*Cox proportional hazard models were used to estimate the individual association of lifestyle and genetic factors with incident cognitive impairment, adjusted for age, sex, entry time, educational attainment, area of residence, current marital status, occupation, and source of income.

#Competing risk analyses were used to test the influence of death as a competing risk for cognitive impairment with adjustment for age, sex, entry time, educational attainment, area of residence, current marital status, occupation, and source of income.

Two-sided *P* <0.05 was considered statistically significant with no correction for multiple comparisons.

Supplementary Table 3. Joint association between genetic risk, lifestyle, and risk of cognitive impairment

Subgroup	Total No. of participants	No. of cases of cognitive impairment/person-years	Cox proportional hazard* models		Competing risk method based on Cox model#	
			Hazard ratio (95% CI)	*P* value	Hazard ratio (95% CI)	*P* value
Low genetic risk						
Favorable lifestyle	508	87/4396	Reference		Reference	
Intermediate lifestyle	1096	272/9122	1.14 (0.89–1.45)	0.293	1.13 (0.89–1.44)	0.320
Unfavorable lifestyle	1546	447/11913	1.35 (1.07–1.70)	0.013	1.29 (1.02–1.63)	0.033
High genetic risk						
Favorable lifestyle	536	96/4560	1.08 (0.81–1.45)	0.583	1.06 (0.80–1.40)	0.699
Intermediate lifestyle	1050	291/8556	1.36 (1.07–1.73)	0.012	1.31 (1.03–1.67)	0.026
Unfavorable lifestyle	1565	463/12162	1.41 (1.11–1.78)	0.004	1.34 (1.06–1.69)	0.014

CI confidence interval.

*Cox proportional hazard models were used to estimate the joint association of lifestyle and genetic factors with incident cognitive impairment, adjusted for age, sex, entry time, educational attainment, area of residence, current marital status, occupation, and source of income.

#Competing risk analyses were used to test the influence of death as a competing risk for cognitive impairment with adjustment for age, sex, entry time, educational attainment, area of residence, current marital status, occupation, and source of income.

Two-sided P <0.05 was considered statistically significant with no correction for multiple comparisons.

Supplementary Table 4. Risk of cognitive impairment according to lifestyle categories within each genetic risk group

Subgroup	Total No. of participants	No. of cases of cognitive impairment/person-years	Cox proportional hazard models*			Competing risk method based on Cox model#		
			Hazard ratio (95% CI)	*P* value	*P* interaction	Hazard ratio (95% CI)	*P* value	*P* interaction
Low genetic risk					0.016			0.064
Unfavorable lifestyle	1546	447/11913	Reference			Reference		
Intermediate lifestyle	1096	272/9122	0.83 (0.71–0.97)	0.018		0.85 (0.72–1.00)	0.05	
Favorable lifestyle	508	87/4396	0.74 (0.58–0.94)	0.014		0.76 (0.60–0.97)	0.028	
High genetic risk								
Unfavorable lifestyle	1565	463/12162	Reference			Reference		
Intermediate lifestyle	1050	291/8556	0.98 (0.84–1.13)	0.75		0.99 (0.85–1.17)	0.98	
Favorable lifestyle	536	96/4560	0.78 (0.62–0.98)	0.033		0.81 (0.64–1.01)	0.065	

CI confidence interval.

*Cox proportional hazard models were used to estimate the association of lifestyle with incident cognitive impairment within each genetic risk group, adjusted for age, sex, entry time, educational attainment, area of residence, current marital status, occupation, and source of income.

#Competing risk analyses were used to test the influence of death as a competing risk for cognitive impairment with adjustment for age, sex, entry time, educational attainment, area of residence, current marital status, occupation, and source of income.

Two-sided *P* <0.05 was considered statistically significant with no correction for multiple comparisons.

Supplementary Table 5. Estimated change in cognitive function by trajectories of lifestyle score

Trajectories of lifestyle score	Standard deviation units			Difference, %
	Estimate (95% CI)	Difference (95% CI)	*P* value	
Cognitive score				
Low–stable	–0.402 (–0.428, –0.376)	Reference		Reference
Intermediate–stable	–0.368 (–0.381, –0.355)	0.034 (0.005, 0.063)	0.02	8.46
High–stable	–0.223 (–0.242, –0.205)	0.179 (0.147, 0.210)	<0.001	44.53
Orientation score				
Low–stable	–0.402 (–0.433, –0.370)	Reference		Reference
Intermediate–stable	–0.368 (–0.384, –0.352)	0.033 (–0.002, 0.069)	0.064	8.21
High–stable	–0.221 (–0.244, –0.199)	0.180 (0.142, 0.219)	<0.001*	44.78
Attention and calculation score				
Low–stable	–0.145 (–0.157, –0.133)	Reference		Reference
Intermediate–stable	–0.131 (–0.137, –0.125)	0.015 (0.001, 0.028)	0.034	10.34
High–stable	–0.081 (–0.089, –0.072)	0.064 (0.050, 0.079)	<0.001*	44.14
Visual construction score				
Low–stable	–0.014 (–0.021, –0.006)	Reference		Reference
Intermediate–stable	–0.015 (–0.019, –0.012)	–0.002 (–0.010, 0.007)	0.675	NA
High–stable	–0.004 (–0.009, 0.001)	0.010 (0.000, 0.019)	0.04	71.43
Language score				
Low–stable	–0.349 (–0.374, –0.324)	Reference		Reference
Intermediate–stable	–0.305 (–0.317, –0.292)	0.044 (0.016, 0.072)	0.002*	12.61
High–stable	–0.174 (–0.192, –0.157)	0.174 (0.144, 0.205)	<0.001*	49.86
Naming score				
Low–stable	–0.166 (–0.182, –0.151)	Reference		Reference
Intermediate–stable	–0.146 (–0.153, –0.138)	0.020 (0.003, 0.038)	0.02	12.05
High–stable	–0.082 (–0.092, –0.071)	0.084 (0.066, 0.103)	<0.001*	50.6
Recall score				
Low–stable	–0.165 (–0.180, –0.151)	Reference		Reference
Intermediate–stable	–0.149 (–0.156, –0.142)	0.016 (0.000, 0.032)	0.044	9.7
High–stable	–0.088 (–0.098, –0.078)	0.078 (0.060, 0.095)	<0.001*	47.27

CI confidence interval.

Linear mixed-effects models were used with adjustment for age, sex, entry time, educational attainment, area of residence, current marital status, occupation, source of income, and baseline cognitive score. For the analysis of cognitive dimensions, models were additionally adjusted for the baseline dimensions of cognitive score as appropriate instead of baseline cognitive score. Two-sided *P* <0.05 was considered statistically significant, except separate analysis for individual domains of cognition in which the Bonferroni correction was applied to account for multiple testing (*P* <0.008 considered significant [= 0.05/6]). *Indicating statistically significant Bonferroni corrected *P* value (*P* < 0.008).

Supplementary Table 6. Components of healthy lifestyle score

Lifestyle factors	Category	Score
Smoking status	Current non-smoking (never smoking, former smoking)	1
	Others	0
Alcohol drinking status	Never alcohol drinking	1
	Others	0
Physical activity*		
Regular exercises	Yes	2
	No	0
Housework tasks, Personal outdoor activities, Gardening, Rearing domestic animals/pets, Reading, Playing cards/mahjong, Watching TV/listening to the radio, Attending social activities	Almost everyday/Not every day, but at least once a week	2
	Not every week, but at least once a month	1
	Not every month, but sometimes/Never	0
Diet intake#		
Fresh vegetables consumption, Fresh fruit consumption	Everyday or almost everyday/Quite often	2
	Occasionally	1
	Rarely or never	0
Legumes consumption, Meat consumption, Egg consumption, Fish and seafood consumption, Tea consumption, Garlic consumption	Almost everyday/Not every day, but at least once per week	2
	Not every week, but at least once per month	1
	Not every month, but occasionally/Rarely or never	0
Salty vegetables consumption	Almost everyday/Not every day, but at least once per week	0
	Not every week, but at least once per month	1
	Not every month, but occasionally/Rarely or never	2

*#Physical activity score and diet score both ranged from 0 to 18. Ideal physical activity/diet intake was defined as the top 40% of the population distribution in line with previous studies. A healthy lifestyle score was constructed based on aforementioned 4 lifestyle factors, ranging from 0 to 4, with higher scores indicating higher adherence to a favorable lifestyle.

Supplementary Table 7. Components of Mini-Mental State Examination

Dimensions	Questions	Maximum Score
Orientation	What is the year?	1
	What is the month?	1
	When is Mid-Autumn Festival?	1
	What season of the year is it?	1
	What county, city or town are we in?	1
Attention and calculation	20-3=?	1
	20-3-3=?	1
	20-3-3-3=?	1
	20-3-3-3-3=?	1
	20-3-3-3-3-3=?	1
Visual construction	Copy the design shown.	1
Language	Can name a ‘pencil’?	1
	Can name a ‘watch’?	1
	Repeat a sentence.	1
	Takes paper in right hand.	1
	Folds paper in half.	1
	Puts paper down on lap.	1
Naming	Naming as many kinds of food as possible in one minute.	7
Recall	Remembered ‘table’?	1
	Remembered ‘apple’?	1
	Remembered ‘clothes’?	1
	Can repeat ‘table’ immediately?	1
	Can repeat ‘apple’ immediately?	1
	Can repeat ‘clothes’ immediately?	1

Supplementary Table 8. List of SNPs included in the polygenic risk score

SNP #	Chromosome	Chromosome position	Gene	Risk Allele	Beta	*P* value
rs6656401	1	207518704	*CR1*	A	0.1655	6.00E-24
rs6701713	1	207612944	*CR1*	A	0.1484	5.00E-10
rs11889338	2	17246318	*ZFYVE9P2, RN7SKP168*	A	0.4383	9.00E-06
rs7561528	2	127132061	*NIFKP9, BIN1*	A	0.157	4.00E-14
rs6733839	2	127135234	*NIFKP9, BIN1*	T	0.1989	7.00E-44
rs1552244	3	10093893	*FANCD2, FANCD2OS*	A	0.2776	2.00E-06
rs6448799	4	11628425	*HS3ST1, LINC02360*	T	0.077	7.00E-08
rs727153	4	154733269	*LRAT*	C	0.4886	3.00E-06
rs190982	5	88927603	*MEF2C-AS1*	A	0.077	3.00E-08
rs9271192	6	32610753	*HLA-DRB1, HLA-DQA1*	C	0.1044	3.00E-12
rs9381040	6	41186912	*TREM2, TREML2*	C	0.077	6.00E-07
rs6922617	6	41368363	*NCR2, FOXP4-AS1*	A	0.09	4.00E-08
rs9349407	6	47485642	*CD2AP*	C	0.1044	9.00E-09
rs10948363	6	47520026	*CD2AP*	G	0.0953	5.00E-11
rs1476679	7	100406823	*ZCWPW1*	T	0.0953	6.00E-10
rs11771145	7	143413669	*EPHA1-AS1*	G	0.1044	1.00E-13
rs10273775	7	147200311	*CNTNAP2*	G	0.4187	9.00E-06
rs28834970	8	27337604	*PTK2B*	C	0.0953	7.00E-14
rs9331896	8	27610169	*CLU*	T	0.1484	3.00E-25
rs7818382	8	95041772	*NDUFAF6*	T	0.0677	8.00E-08
rs956225	8	121897448	*HAS2-AS1*	A	1.203	9.00E-06
rs514716	9	3929424	*GLIS3*	G	0.071	3.00E-09
rs7920721	10	11678309	*USP6NL-AS1, ECHDC3*	G	0.0677	3.00E-07
rs474951	11	60071148	*MS4A3, MS4A2*	T	0.239	1.00E-06
rs10792832	11	86156833	*RNU6-560P, LINC02695*	G	0.1398	9.00E-26
rs17511627	13	26150190	*ATP8A2P3, RNF6*	C	0.5596	5.00E-06
rs17125944	14	52933911	*FERMT2*	C	0.131	8.00E-09
rs10498633	14	92460608	*SLC24A4*	G	0.0953	6.00E-09
rs3752246	19	1056493	*ABCA7*	G	0.1398	6.00E-07
rs4147929	19	1063444	*ABCA7*	A	0.1398	1.00E-15
rs6859	19	44878777	*NECTIN2*	A	0.3436	1.00E-07
rs8035452	15	50748601	*SPPL2A*	T	0.077	3.00E-07
rs429358*	19	44908684	*APOE*	T		
rs7412*	19	44908822	*APOE*	C		

SNP single nucleotide polymorphisms.

#All the selected SNPs were associated with Alzheimer's disease. **APOE ε2* and *ε4* allele were obtained based on the combination of *APOE* rs429358 and *APOE* rs7412 polymorphisms, with the effect value for Alzheimer's disease of -0.47 and 1.03, respectively. The weight coefficient and *P* value for each SNP was reported in previous studies [17] [18].

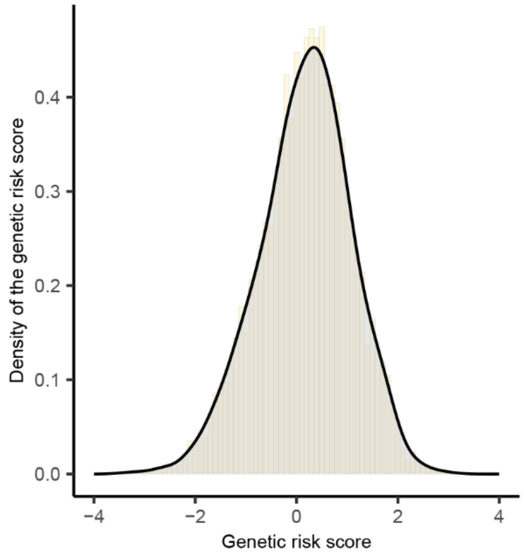

Supplementary Figure 1. Distribution of polygenic risk score of cognitive function. Source data are provided as a Source Data file.

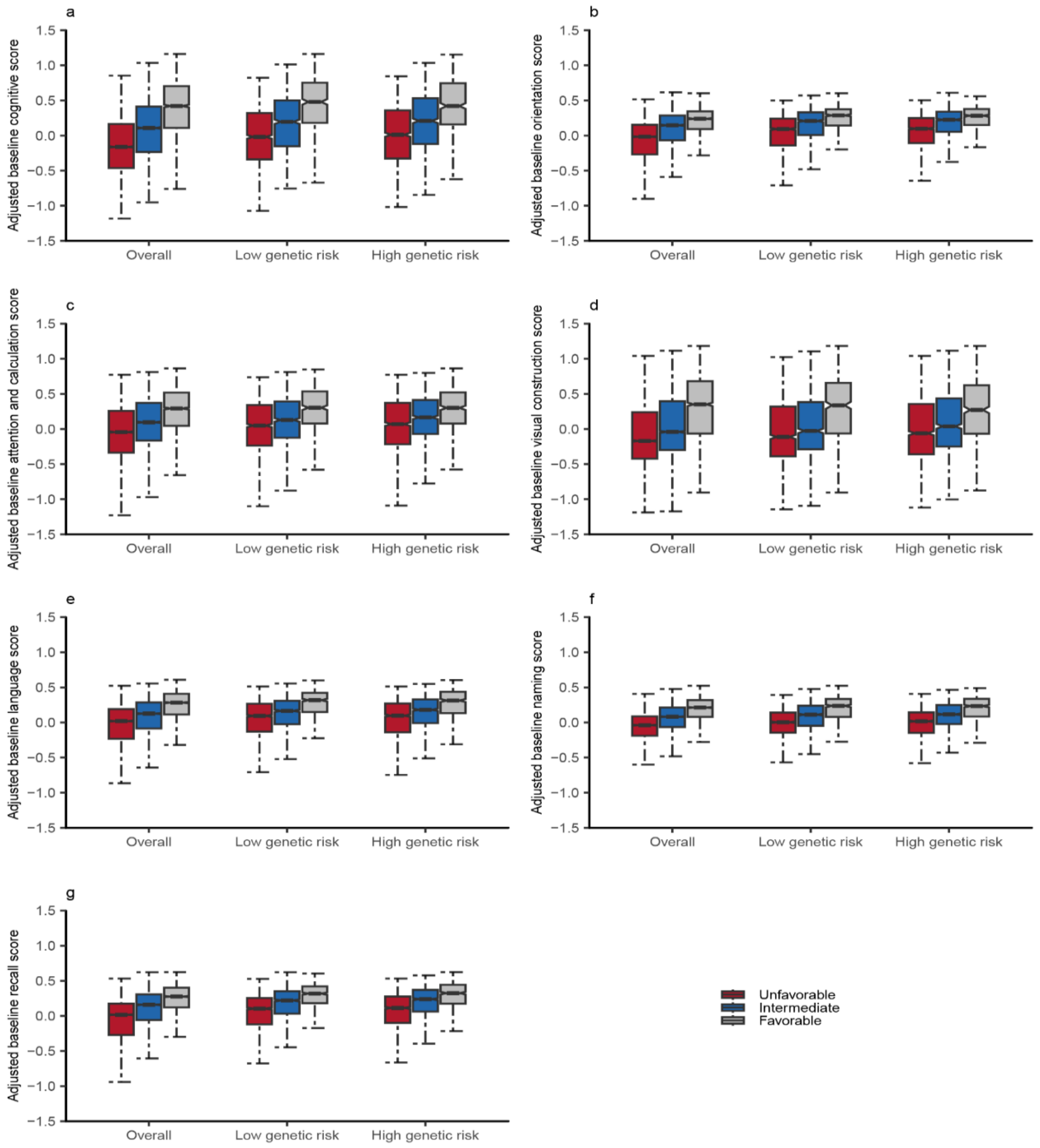

Supplementary Figure 2. Adjusted values of baseline cognitive score among overall participants and different genetic risk groups according to lifestyle categories. Multiple linear regression models were used to estimate the marginal mean values for cognitive score and its individual dimensions according to lifestyle categories, with adjustment for age, sex, entry time, educational attainment, area of residence, current marital status, occupation, source of income, baseline cognitive score or the other dimensions of baseline cognitive score where appropriate. a) for overall cognitive score, b) for the orientation score, c) for the attention and calculation score, d) for the visual construction score, e) for the language score, f) for the naming score, and g) for the recall score. Box plots represents lifestyle type, with the center line indicating the median, the box bounds representing the 25 and 75 percentiles of adjusted baseline cognitive score, and the whiskers extending to minima and maxima, 1.5 times the interquartile range from the 25th and 75th percentiles, respectively. Source data are provided as a Source Data file.

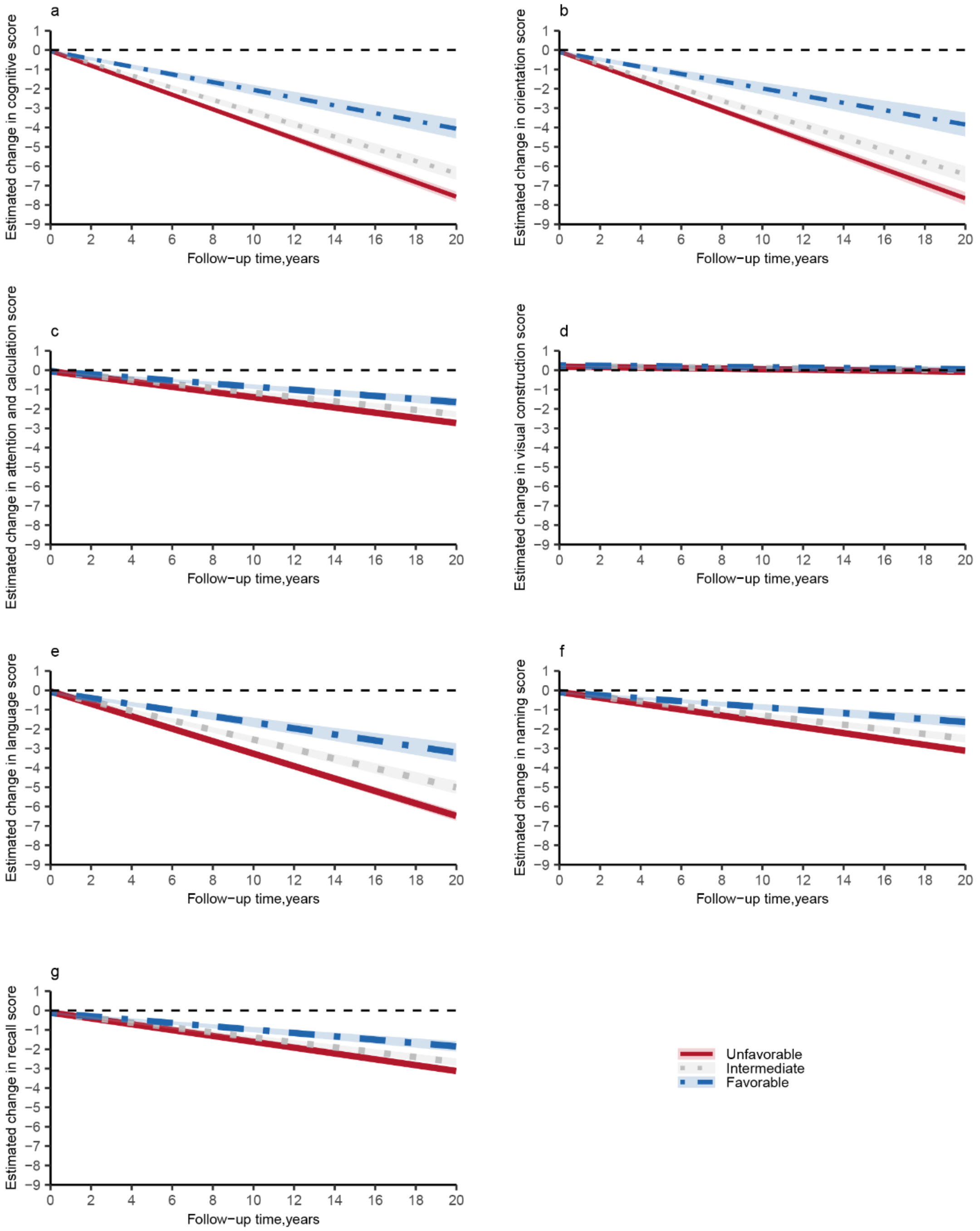

Supplementary Figure 3. Rate of change in cognitive score among overall participants according to lifestyle categories. Linear mixed-effects models were used with adjustment for age, sex, entry time, educational attainment, area of residence, current marital status, occupation, source of income, and baseline cognitive score. For dimensions of cognitive function, models were further adjusted for the other dimensions of baseline cognitive score where appropriate. a) for overall cognitive score, b) for the orientation score, c) for the attention and calculation score, d) for the visual construction score, e) for the language score, f) for the naming score, and g) for the recall score. Solid red line represents points estimates of unfavorable lifestyle group, grey dotted line represents points estimates of intermediate lifestyle

group, and blue dashed line represents points estimates of favorable lifestyle group. The shaded areas show 95% confidence interval (CI) of estimated change in cognitive score. Source data are provided as a Source Data file.

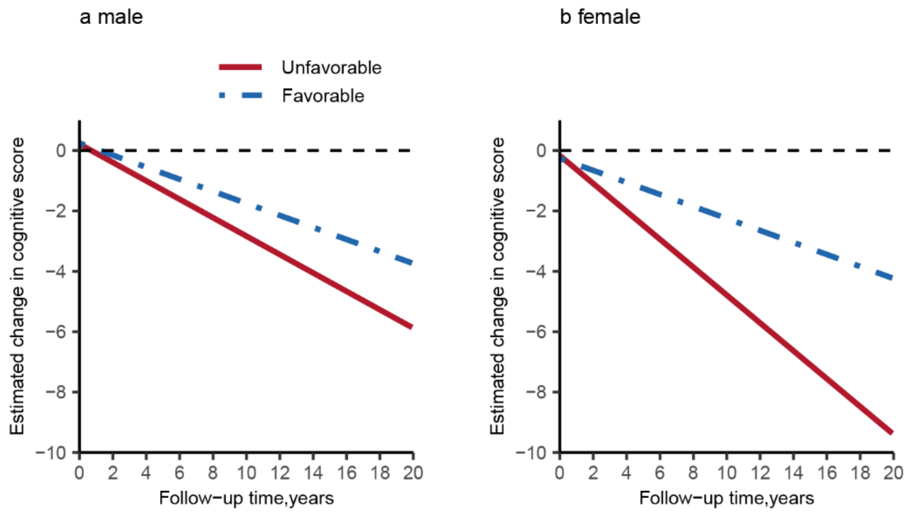

Supplementary Figure 4. Association of healthy lifestyle with cognitive decline stratified by sex. Linear mixed-effects models were used with adjustment for age, entry time, educational attainment, area of residence, current marital status, occupation, source of income, and baseline cognitive score. a) among male, the association of lifestyle with cognitive decline, and b) among female, the association of lifestyle with cognitive decline. Solid red line represents points estimates of unfavorable lifestyle group, blue dashed line represents points estimates of favorable lifestyle group. Source data are provided as a Source Data file.

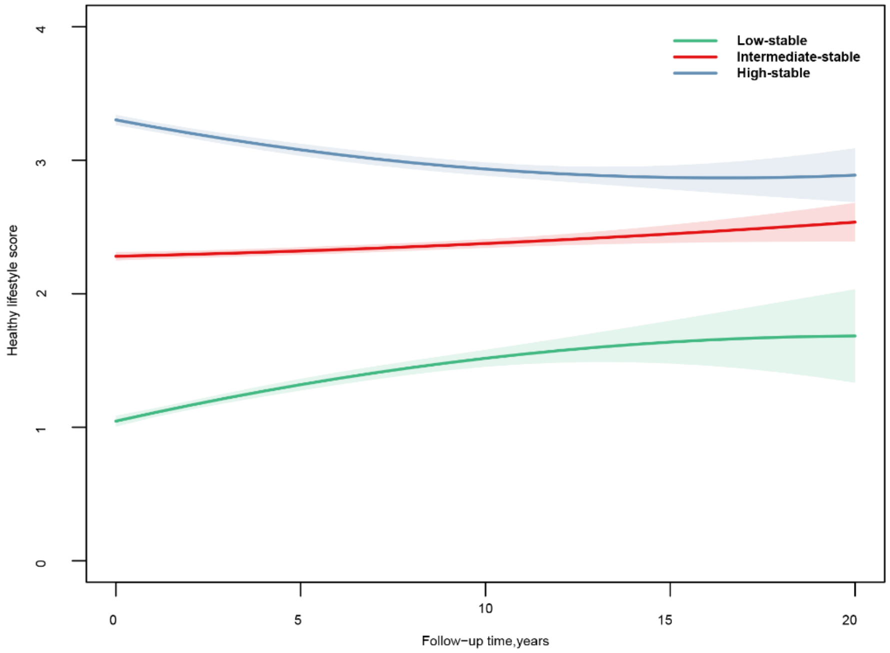

Supplementary Figure 5. Trajectories of healthy lifestyle score for older adults during follow-up. Latent class trajectory modeling was used to identify distinct lifestyle score trajectories. Solid lines represent the estimates of the healthy lifestyle score trajectories and shaded areas represent the 95% confidence interval (CI). Source data are provided as a Source Data file.

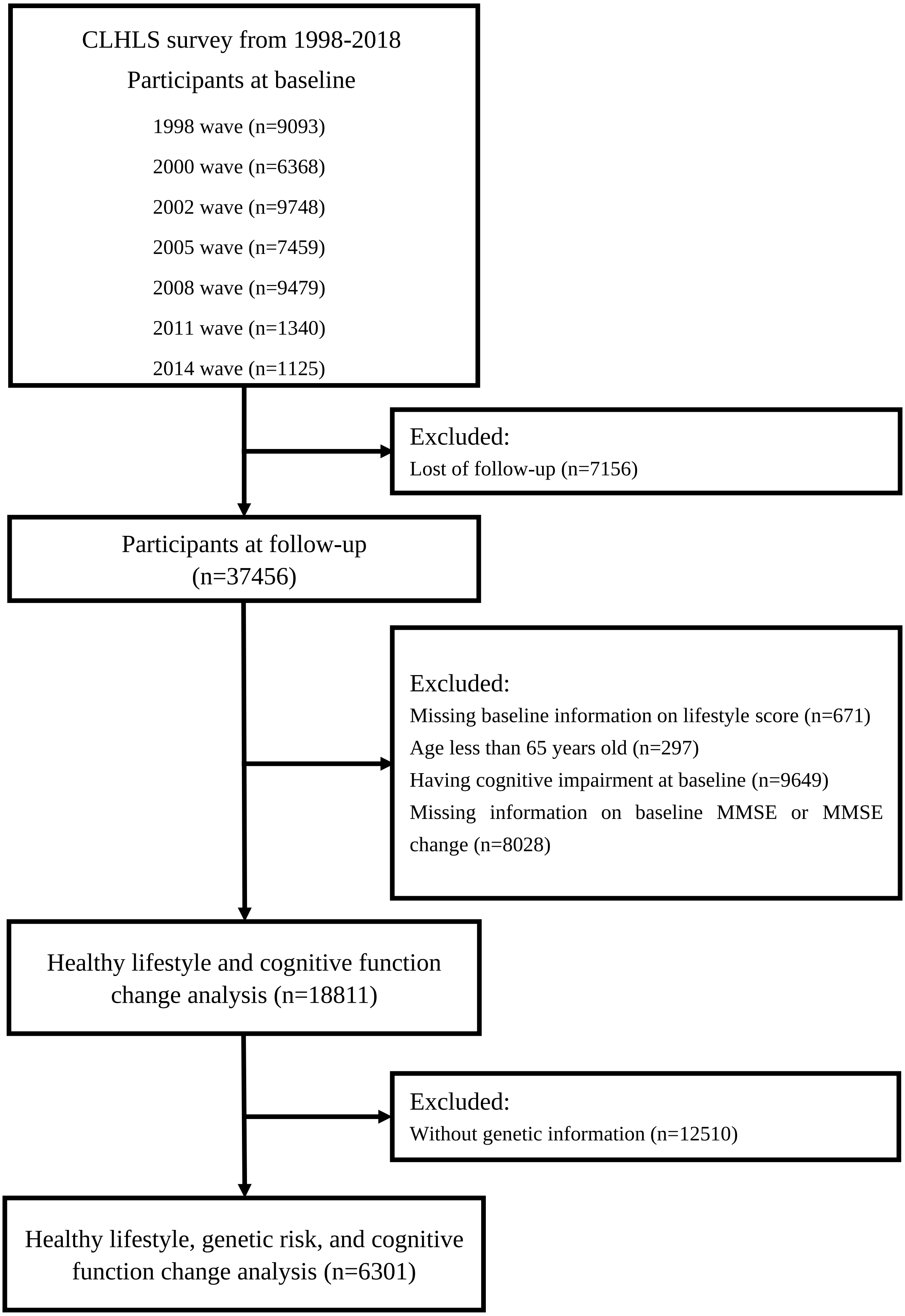

Supplementary Figure 6. A participant flow chart

Supplementary References:

1. Dhana K, *et al.* Genetic risk, adherence to a healthy lifestyle, and cognitive decline in African Americans and European Americans. *Alzheimers Dement* **18**, 572-580 (2022).
2. Jia J, *et al.* Association between healthy lifestyle and memory decline in older adults: 10 year, population based, prospective cohort study. *BMJ (Clinical research ed)* **380**, e072691 (2023).
3. Zhu A, *et al.* Interaction between plant-based dietary pattern and air pollution on cognitive function: a prospective cohort analysis of Chinese older adults. *Lancet Reg Health West Pac* **20**, 100372 (2022).
4. Zeng Y, Gu D, Purser J, Hoenig H, Christakis N. Associations of environmental factors with elderly health and mortality in China. *Am J Public Health* **100**, 298-305 (2010).
5. Su S, *et al.* Leisure Activities and the Risk of Dementia: A Systematic Review and Meta-Analysis. *Neurology* **99**, e1651-1663 (2022).
6. Li ZH, *et al.* Leisure Activities and All-Cause Mortality Among the Chinese Oldest-Old Population: A Prospective Community-Based Cohort Study. *Journal of the American Medical Directors Association* **21**, 713-719.e712 (2020).
7. Zhang Y, Fu S, Ding D, Lutz MW, Zeng Y, Yao Y. Leisure Activities, APOE ε4, and Cognitive Decline: A Longitudinal Cohort Study. *Front Aging Neurosci* **13**, 736201 (2021).
8. Chen X, *et al.* Mediation analysis of leisure activities on the association between cognitive function and mortality: a longitudinal study of 42,942 Chinese adults 65 years and older. *Epidemiol Health* **44**, e2022112 (2022).
9. Zhang YB, *et al.* Associations of healthy lifestyle and socioeconomic status with mortality and incident cardiovascular disease: two prospective cohort studies. *BMJ (Clinical research ed)* **373**, n604 (2021).
10. Chen H, Zhang X, Feng Q, Zeng Y. The Effects of "Diet-Smoking-Gender" Three-Way Interactions on Cognitive Impairment among Chinese Older Adults. *Nutrients* **14**, 2144 (2022).
11. Yan LL, *et al.* Healthy eating and all-cause mortality among Chinese aged 80 years or older. *Int J Behav Nutr Phys Act* **19**, 60 (2022).
12. Zhang Y, *et al.* Interaction between APOE epsilon4 and dietary protein intake on cognitive decline: A longitudinal cohort study. *Clinical nutrition (Edinburgh, Scotland)* **40**, 2716-2725 (2021).
13. Kim H, Lee K, Rebholz CM, Kim J. Plant-based diets and incident metabolic syndrome: Results from a South Korean prospective cohort study. *PLoS Med* **17**, e1003371 (2020).
14. Pan XF, Li Y, Franco OH, Yuan JM, Pan A, Koh WP. Impact of Combined Lifestyle Factors on All-Cause and Cause-Specific Mortality and Life Expectancy in Chinese: The Singapore Chinese Health Study. *The journals of gerontology Series A, Biological sciences and medical sciences* **75**, 2193-2199

(2020).
15. Xi D, *et al.* Risk factors associated with heatwave mortality in Chinese adults over 65 years. *Nat Med*, **30**, 1489-1498 (2024).
16. Zhang PD, *et al.* Age, Period, and Cohort Effects on Activities of Daily Living, Physical Performance, and Cognitive Functioning Impairment Among the Oldest-Old in China. *The journals of gerontology Series A, Biological sciences and medical sciences* **75**, 1214-1221 (2020).
17. Liu X, *et al.* Integrated genetic analyses revealed novel human longevity loci and reduced risks of multiple diseases in a cohort study of 15,651 Chinese individuals. *Aging Cell* **20**, e13323 (2021).
18. Jin X, Shu C, Zeng Y, Liang L, Ji JS. Interaction of greenness and polygenic risk score of Alzheimer's disease on risk of cognitive impairment. *The Science of the total environment* **796**, 148767 (2021).
