# Supplementary material for: Integrated healthy lifestyle even in late-life mitigates cognitive decline risk across varied genetic susceptibility
Source: Nat Commun. 2025 Jan 9;16:539. doi: 10.1038/s41467-024-55763-0 (PMC11718162; doi:10.1038/s41467-024-55763-0)
Supplement: Supplementary file 2 — Description of Additional Supplementary Files [file 41467_2024_55763_MOESM2_ESM.pdf]

Description of Additional Supplementary Files

File Name: Supplementary Data 1

Description: Baseline characteristics of participants by lifestyle and genetic risk groups. Mean (standard deviation, SD) for continuous variables and number (percentage) for dichotomous variables. Differences between groups were compared with analysis of variance or chi-square test. Two-sided P <0.05 was considered statistically significant. No correction for multiple comparisons applied.

File Name: Supplementary Data 2

Description: Longitudinal cognitive decline according to genetic and lifestyle categories. Participants were divided into low genetic risk and high genetic risk group. Linear mixed-effects models were used with adjustment for age, sex, entry time, educational attainment, area of residence, current marital status, occupation, source of income, and baseline cognitive score. For the analysis of six cognitive dimensions, models were additionally adjusted for the baseline dimensions of cognitive score as appropriate instead of baseline cognitive score. Two-sided P <0.05 was considered statistically significant, except separate analysis for individual domains of cognition in which the Bonferroni correction was applied to account for multiple testing (P <0.008 considered significant [= 0.05/6]).

File Name: Supplementary Data 3

Description: Joint effect of genetic risk and lifestyle factors on rate of cognitive decline. Participants were categorized into six groups according to the different combinations of genetic risk (low, high), and lifestyle (unfavorable, intermediate, and favorable). Linear mixed-effects models were used with adjustment for age, sex, entry time, educational attainment, area of residence, current marital status, occupation, source of income, and baseline cognitive score. For the analysis of six cognitive dimensions, models were additionally adjusted for the baseline dimensions of cognitive score as appropriate instead of baseline cognitive score. Two-sided P <0.05 was considered statistically significant, except separate analysis for individual domains of cognition in which the Bonferroni correction was applied to account for multiple testing (P <0.008 considered significant [= 0.05/6]).

File Name: Supplementary Data 4

Description: Sensitivity analyses for association of healthy lifestyle with cognitive decline among overall participants. a) Further adjusted for self-reported health status. Linear mixed-effects models were used, and adjusted for age, sex, entry time, educational attainment, area of residence, current marital status, occupation, source of income, baseline cognitive score, and self-reported health status. For the analysis of six cognitive dimensions, models were additionally adjusted for the baseline dimensions of cognitive score as appropriate instead of baseline cognitive score. b) Further adjusted for optimism status. Linear mixed-effects models were used, and adjusted for age, sex, entry time, educational attainment, area of residence, current

marital status, occupation, source of income, baseline cognitive score, and optimism status. For the analysis of six cognitive dimensions, models were additionally adjusted for the baseline dimensions of cognitive score as appropriate instead of baseline cognitive score. c) Further adjusted for chronic disease status. Linear mixed-effects models were used, and adjusted for age, sex, entry time, educational attainment, area of residence, current marital status, occupation, source of income, baseline cognitive score, and chronic disease status. For the analysis of six cognitive dimensions, models were additionally adjusted for the baseline dimensions of cognitive score as appropriate instead of baseline cognitive score. d) Excluding participants with baseline MMSE in the lowest 10% of the cohort distribution; e) Excluding participants with cognitive score change at the 0.5^{th} and 99.5^{th} percentiles of the cohort distribution; f) Using "never smoking" as a healthy lifestyle factor instead of "no current smoking" status to develop a new healthy lifestyle score; g) Participants who completed all items of MMSE test. Linear mixed-effects models were used in d), e), f), g), and adjusted for age, sex, entry time, educational attainment, area of residence, current marital status, occupation, source of income, and baseline cognitive score. For the analysis of six cognitive dimensions, models were additionally adjusted for the baseline dimensions of cognitive score as appropriate instead of baseline cognitive score. Two-sided P <0.05 was considered statistically significant, except separate analysis for individual domains of cognition in which the Bonferroni correction was applied to account for multiple testing (P <0.008 considered significant [= 0.05/6]).

File Name: Supplementary Data 5
Description: Sensitivity analyses for association of healthy lifestyle with cognitive decline in different genetic risk groups. a) Further adjusted for self-reported health status. Linear mixed-effects models were used, and adjusted for age, sex, entry time, educational attainment, area of residence, current marital status, occupation, source of income, baseline cognitive score, and self-reported health status. For the analysis of six cognitive dimensions, models were additionally adjusted for the baseline dimensions of cognitive score as appropriate instead of baseline cognitive score. b) Further adjusted for optimism status. Linear mixed-effects models were used, and adjusted for age, sex, entry time, educational attainment, area of residence, current marital status, occupation, source of income, baseline cognitive score, and optimism status. For the analysis of six cognitive dimensions, models were additionally adjusted for the baseline dimensions of cognitive score as appropriate instead of baseline cognitive score. c) Further adjusted for chronic disease status. Linear mixed-effects models were used, and adjusted for age, sex, entry time, educational attainment, area of residence, current marital status, occupation, source of income, baseline cognitive score, and chronic disease status. For the analysis of six cognitive dimensions, models were additionally adjusted for the baseline dimensions of cognitive score as appropriate instead of baseline cognitive score. d) Excluding participants with baseline MMSE in the lowest 10% of the cohort distribution; e) Excluding participants with cognitive score change at the 0.5^{th} and 99.5^{th} percentiles of the

cohort distribution; f) Using “never smoking” as a healthy lifestyle factor instead of “no current smoking” status to develop a new healthy lifestyle score; g) Participants who completed all items of MMSE test. Linear mixed-effects models were used in d), e), f), g), and adjusted for age, sex, entry time, educational attainment, area of residence, current marital status, occupation, source of income, and baseline cognitive score. For the analysis of six cognitive dimensions, models were additionally adjusted for the baseline dimensions of cognitive score as appropriate instead of baseline cognitive score. Two-sided P <0.05 was considered statistically significant, except separate analysis for individual domains of cognition in which the Bonferroni correction was applied to account for multiple testing (P <0.008 considered significant [= 0.05/6]).
