# Supplementary material for: Integrated healthy lifestyle even in late-life mitigates cognitive decline risk across varied genetic susceptibility
Source: Nat Commun. 2025 Jan 9;16:539. doi: 10.1038/s41467-024-55763-0 (PMC11718162; doi:10.1038/s41467-024-55763-0)
Supplement: Supplementary file 8 — Reporting Summary [file 41467_2024_55763_MOESM8_ESM.pdf]

nature portfolio

Corresponding author(s): Xiaoming Shi

Last updated by author(s): Dec 19, 2024

Reporting Summary

Nature Portfolio wishes to improve the reproducibility of the work that we publish. This form provides structure for consistency and transparency in reporting. For further information on Nature Portfolio policies, see our Editorial Policies and the Editorial Policy Checklist.

Statistics

For all statistical analyses, confirm that the following items are present in the figure legend, table legend, main text, or Methods section.

n/a	Confirmed	
☐	☒	The exact sample size (*n*) for each experimental group/condition, given as a discrete number and unit of measurement
☐	☒	A statement on whether measurements were taken from distinct samples or whether the same sample was measured repeatedly
☐	☒	The statistical test(s) used AND whether they are one- or two-sided<br>*Only common tests should be described solely by name; describe more complex techniques in the Methods section.*
☐	☒	A description of all covariates tested
☐	☒	A description of any assumptions or corrections, such as tests of normality and adjustment for multiple comparisons
☐	☒	A full description of the statistical parameters including central tendency (e.g. means) or other basic estimates (e.g. regression coefficient) AND variation (e.g. standard deviation) or associated estimates of uncertainty (e.g. confidence intervals)
☐	☒	For null hypothesis testing, the test statistic (e.g. *F*, *t*, *r*) with confidence intervals, effect sizes, degrees of freedom and *P* value noted<br>*Give P values as exact values whenever suitable.*
☒	☐	For Bayesian analysis, information on the choice of priors and Markov chain Monte Carlo settings
☒	☐	For hierarchical and complex designs, identification of the appropriate level for tests and full reporting of outcomes
☐	☒	Estimates of effect sizes (e.g. Cohen's *d*, Pearson's *r*), indicating how they were calculated

Our web collection on statistics for biologists contains articles on many of the points above.

Software and code

Policy information about availability of computer code

Data collection	Data collection was carried out by paper questionnaire. Questionnaire data was input by Epidata via double data entry.
Data analysis	Statistical analysis was done using SAS 9.4 Software License Renewal Order 9CWMQY (Tech Sup # 70308905), and R V.4.3.1 (R Foundation for Statistical Computing, Vienna, Austria).

For manuscripts utilizing custom algorithms or software that are central to the research but not yet described in published literature, software must be made available to editors and reviewers. We strongly encourage code deposition in a community repository (e.g. GitHub). See the Nature Portfolio guidelines for submitting code & software for further information.

Data

Policy information about availability of data

All manuscripts must include a data availability statement. This statement should provide the following information, where applicable:

- Accession codes, unique identifiers, or web links for publicly available datasets
- A description of any restrictions on data availability
- For clinical datasets or third party data, please ensure that the statement adheres to our policy

All CLHLS data used in this study, except for the raw genetic sequencing data, are available from the official repository located at https://doi.org/10.18170/DVN/WBO7LK. The raw genetic sequencing data of CLHLS used in this study have been deposited and controlled at the CNGB Sequence Archive (CNSA, https://db.cngb.org/cnsa/) of the China National GeneBank DataBase (CNGBdb) under accession code CNP0000792 (https://db.cngb.org/search/?q=CNP0000792). The raw

genetic sequencing data used in this study are available under restricted access due to data privacy laws and ethical restrictions. Access can be obtained by completing the application form via https://db.cngb.org/data_access/ or by contacting the corresponding authors (shixm@chinacdc.cn or lvyuebin@nieh.chinacdc.cn), and will be answered within 10 weeks. The sharing requests for raw genetic sequencing data of CLHLS will be reviewed by the institutional ethical committees of CLHLS study to verify whether the request is subject to any intellectual property or confidentiality obligations. This study did not generate new genetic sequencing data. All other data supporting the findings described in this study are available in the article, Supplementary Information, and Source Data files. Source data are provided with this paper.

Research involving human participants, their data, or biological material

Policy information about studies with human participants or human data. See also policy information about sex, gender (identity/presentation), and sexual orientation and race, ethnicity and racism.

Reporting on sex and gender	We have carefully used the term sex to avoid confusion with gender. Sex data were self-reported using structured questions, with the options “female” or “male” provided. We present sex-based analyses.
Reporting on race, ethnicity, or other socially relevant groupings	Self-reported information on race/ethnicity (Han Chinese, others), educational attainment (years of schooling), and source of income (independent, dependent) was collected by face-to-face interview.
Population characteristics	Over eight waves from 1998 to 2018, the Chinese Longitudinal Healthy Longevity Survey (CLHLS) conducted 113 thousands face-to-face interviews with 19.5 thousand centenarians, 26.7 thousands nonagenarians, 29.7 thousands octogenarians, 25.5 thousands younger elders aged 65-79, and 11.3 thousands middle-age adults aged 35-64. For participants aged 65-118 who died between waves, data on mortality and degree/length of disability before death were collected in interviews with a close family member of the deceased. All of the interviews, physical exams and biological specimens collections are voluntary with standard consensus forms reviewed/signed by the participants (or their direct family members).
Recruitment	CLHLS is a dynamic cohort study, launched in 1998 and a total of 7 times follow-up interviews were conducted in 2000, 2002, 2005, 2008/2009, 2011/2012, 2014 and 2018. Participants were recruited from a randomly selected half of the counties and cities in 23 out of 31 provinces in China. Deceased participants were substituted by new participants of the same gender and within the same age range of five years.
Ethics oversight	The CLHLS received approval from the Ethics Committee of Peking University (IRB00001052-13074). Written informed consent was obtained from all participants or their legal representatives during the face-to-face interview.

Note that full information on the approval of the study protocol must also be provided in the manuscript.

Field-specific reporting

Please select the one below that is the best fit for your research. If you are not sure, read the appropriate sections before making your selection.

☐ Life sciences ☒ Behavioural & social sciences ☐ Ecological, evolutionary & environmental sciences

For a reference copy of the document with all sections, see nature.com/documents/nr-reporting-summary-flat.pdf

Behavioural & social sciences study design

All studies must disclose on these points even when the disclosure is negative.

Study description	Our analysis employs a longitudinal cohort design to assess the role of combined lifestyle factors and genetic risk on decline rate of overall cognitive function and specific cognitive domains among Chinese older adults. The data were quantitative.
Research sample	Of 37456 participants completed the baseline and follow-up investigations. We excluded participants with missing information on lifestyle score at baseline (n=671), those aged less than 65 years old (n=297), those with cognitive impairment at baseline (n=9649), and those without available MMSE measurements at baseline or any follow-up surveys (n=8028). Ultimately, 18811 cognitively healthy older adults with average age of 82.97 years from CLHLS participating in at least two waves of investigation were included in the analysis for healthy lifestyle and cognitive decline, and of those, 6301 participants with genotyping data were in included for the joint association between healthy lifestyle and genetic risk analysis.
Sampling strategy	Of the total participants recruited from 8 waves of CLHLS, 37456 participants completed the baseline and follow-up examinations. We further excluded participants with missing information on lifestyle score at baseline (n=671), those aged less than 65 years old (n=297), those with cognitive impairment at baseline (n=9649), and those without available MMSE measurements at baseline or any follow-up surveys (n=8028). Finally, a total of 18811 participants were involved in the current analysis, and of those, 6301 participants with genotyping data were in included for the joint association between healthy lifestyle and genetic risk analysis. The chosen sample sizes are deemed sufficient for our study to explore the association between lifestyle, genetic risk and subsequent cognitive decline. In addition, to test the robustness of our results, we performed several sensitivity analyses. The strength and magnitude of association between the healthy lifestyle with cognitive decline and its respective domains among overall participants and different genetic risk group remained largely unchanged in a series of sensitivity analyses.
Data collection	Data collection was carried out by paper questionnaire. Questionnaire data was input by Epidata via double data entry. The researcher was not blinded to the experimental condition or the study hypothesis.

Timing	The follow-up period of our longitudinal dataset started from Jan 13, 1998 and ended at Jun 8, 2019.
Data exclusions	Of the total recruited from 8 waves of CLHLS, 37456 participants completed the baseline and follow-up examinations. We excluded participants with missing information on lifestyle score at baseline (n=671), those aged less than 65 years old (n=297), those with cognitive impairment at baseline (n=9649), and those without available MMSE measurements at baseline or any follow-up surveys (n=8028). Finally, 18811 participants remained in the current analysis, and of those, 6301 participants with genotyping data were in included for the joint association between healthy lifestyle and genetic risk analysis.
Non-participation	8028 of the participants did not have available MMSE measurements at baseline or any follow-up surveys. For genetic and lifestyle analysis, we additionally excluded 12510 individuals without genotyping information.
Randomization	Not applicable as no intervention was provided to any participant. Covariates were selected based on prior research and available cohort ascertained by self-report, including age, sex, educational level, area of residence, marital status, occupation, source of income, self-reported health status, optimism status, and history of major chronic disease.

Reporting for specific materials, systems and methods

We require information from authors about some types of materials, experimental systems and methods used in many studies. Here, indicate whether each material, system or method listed is relevant to your study. If you are not sure if a list item applies to your research, read the appropriate section before selecting a response.

Materials & experimental systems

n/a	Involved in the study
☒	☐ Antibodies
☒	☐ Eukaryotic cell lines
☒	☐ Palaeontology and archaeology
☒	☐ Animals and other organisms
☒	☐ Clinical data
☒	☐ Dual use research of concern
☒	☐ Plants

Methods

n/a	Involved in the study
☒	☐ ChIP-seq
☒	☐ Flow cytometry
☒	☐ MRI-based neuroimaging

Plants

Seed stocks	Not applicable.
Novel plant genotypes	Not applicable.
Authentication	Not applicable.
